# Inhibition of hyaluronic acid degradation pathway suppresses glioma progression by inducing apoptosis and cell cycle arrest

**DOI:** 10.1186/s12935-023-02998-4

**Published:** 2023-08-11

**Authors:** Tao Yan, He Yang, Caixia Xu, Junsi Liu, Yun Meng, Qing Jiang, Jinxing Li, Guiqiong Kang, Liangjian Zhou, Shuai Xiao, Yanpeng Xue, Jiayi Xu, Xin Chen, Fengyuan Che

**Affiliations:** 1 Central Laboratory, Linyi People’s Hospital, Guangzhou University of Chinese Medicine, Linyi, 276000 Shandong Province China; 2 Linyi Key Laboratory of Neurophysiology, Linyi People’s Hospital, Linyi, 276000 Shandong Province China; 3 Department of Neurosurgery, First Affiliated Hospital of Harbin Medical University, Harbin, 150001 Heilongjiang Province China; 4 Key Colleges and Universities Laboratory of Neurosurgery in Heilongjiang Province, Harbin, 150001 Heilongjiang Province China; 5 Institute of Neuroscience, Sino-Russian Medical Research Center, Harbin Medical University, Harbin, 150001 Heilongjiang Province China; 6 Department of Neurosurgery, Linyi People’s Hospital, Linyi, 276000 Shandong Province China; 7 Scientific Research Management Office, Linyi People’s Hospital, Linyi, 276000 Shandong Province China; 8 Department of Neurology, Linyi People’s Hospital, Linyi, 276000 Shandong Province China

**Keywords:** Glioma, Hyaluronic acid, HYAL2, Apoptosis, Cell cycle

## Abstract

**Background:**

Abnormal hyaluronic acid (HA) metabolism is a major factor in tumor progression, and the metabolic regulation of HA mainly includes HA biosynthesis and catabolism. In glioma, abnormal HA biosynthesis is intimately involved in glioma malignant biological properties and the formation of immunosuppressive microenvironment; however, the role of abnormal HA catabolism in glioma remains unclear.

**Methods:**

HA catabolism is dependent on hyaluronidase. In TCGA and GEPIA databases, we found that among the 6 human hyaluronidases (HYAL1, HYAL2, HYAL3, HYAL4, HYALP1, SPAM1), only HYAL2 expression was highest in glioma. Next, TCGA and CGGA database were further used to explore the correlation of HYAL2 expression with glioma prognosis. Then, the mRNA expression and protein level of HYAL2 was determined by qRT-PCR, Western blot and Immunohistochemical staining in glioma cells and glioma tissues, respectively. The MTT, EdU and Colony formation assay were used to measure the effect of HYAL2 knockdown on glioma. The GSEA enrichment analysis was performed to explore the potential pathway regulated by HYAL2 in glioma, in addition, the HYAL2-regulated signaling pathways were detected by flow cytometry and Western blot. Finally, small molecule compounds targeting HYAL2 in glioma were screened by Cmap analysis.

**Results:**

In the present study, we confirmed that Hyaluronidase 2 (HYAL2) is abnormally overexpressed in glioma. Moreover, we found that HYAL2 overexpression is associated with multiple glioma clinical traits and acts as a key indicator for glioma prognosis. Targeting HYAL2 could inhibit glioma progression by inducing glioma cell apoptosis and cell cycle arrest.

**Conclusion:**

Collectively, these observations suggest that HYAL2 overexpression could promote glioma progression. Thus, treatments that disrupt HA catabolism by altering HYAL2 expression may serve as effective strategies for glioma treatment.

**Supplementary Information:**

The online version contains supplementary material available at 10.1186/s12935-023-02998-4.

## Introduction

Glioma has a very high mortality and disability rate, with an annual incidence of 4.67 to 5.73 per 100,000, and it is also the most common primary intracranial malignant tumor, posing a serious hazard to human health and bringing enormous social pressure [1]. Accumulating evidences suggest that even after surgical resection combined with radiotherapy and chemotherapy, the survival time of patients with this refractory brain tumor is still unsatisfactory [2]. Consequently, novel and effective remedies need to be explored for this disease.

An increasing number of studies have shown that tumor microenvironment remodeling is conducive to unrestricted tumor proliferation, and leads to further modification of malignant tumor behaviors, which are critical for tumor progression [3]. Conceivably, within the tumor, under the action of a distinct tumor microenvironment, tumor cells can selectively produce mutations that engender survival and expansion, lead to tumor heterogeneity, and ultimately promote tumor progression [4]. Similarly, tumor microenvironment remodeling plays a crucial role in glioma progression. Sungho Lee et al. reported that CX3CR1 could alter the tumor microenvironment to enhance tumor-associated macrophages (TAMs) accumulation and angiogenesis during low-grade glioma (LGG) malignant transformation [5]. In the glioma stem cell (GSC) niche, the establishment and remodeling of the tumor microenvironment benefit from the crosstalk between GSCs and other surrounding cells, thereby well-establishing the tumorigenic behavior of GSCs [3]. In glioblastoma (GBM), therapies that modulate tumor angiogenesis and immunosuppressive microenvironments have shown great therapeutic potential [6]. Therefore, further study of the relationship between the tumor microenvironment and glioma cells is expected to reveal the reasons for the poor treatment outcomes and poor prognosis of glioma.

The extracellular matrix (ECM) acts as an important barrier that prevents normal tissue from becoming cancerous. However, the crosstalk of tumor cells with healthy tissues can reshape the extracellular matrix, recruit immune cells, and promote angiogenesis, which converts this environment into a supportive microenvironment for tumor progression [4]. As a major component of the ECM, hyaluronic acid (HA) is considered a main participant in tumor initiation and progression [7]. In addition, abnormal HA metabolism is observed in various tumors. For example, abnormal HA accumulation could alter the malignant phenotype of breast cancer [8]. In prostate cancer cells, HA is upregulated, and 4-methylumbelliferone (4-MU) could exhibit an inhibitory effect on prostate cancer cell proliferation by inhibiting HA synthesis [9]. These reports suggest that the abnormal metabolism of HA plays a crucial role in the regulation of tumor progression. In our previous study, we demonstrated that inhibiting the HA pathway could suppress glioma proliferation in an autophagy-dependent manner, and disturbing glioblastoma HA synthesis could affect M1-like TAM polarization via the STAT1 pathway [10, 11], which further suggested that abnormal HA metabolism also occurs in glioma cells. Moreover, abnormal HA accumulation also participates in the reshaping of the glioma microenvironment [11].

Nevertheless, in our previous reports, we mainly discussed the effect of abnormal HA biosynthesis on malignant glioma behaviors. In the present study, we wanted to elaborate the role of HA catabolism in glioma progression. And HA degradation is mainly mediated by hyaluronidase. Furthermore, some researchers reported that hyaluronidase is associated with tumor malignant progression, which has been demonstrated in prostate cancer, urinary bladder cancers, laryngeal cancer and breast cancer [12, 13]. Therefore, further study of abnormal HA degradation in tumors may reveal an effective strategy for tumor treatment.

In the present study, our results confirmed that among the six hyaluronidases (HYAL1, HYAL2, HYAL3, HYAL4, HYALP1, SPAM1), only HYAL2 was overexpressed in glioma patients, and HYAL2 overexpression was negatively correlated with the survival time of glioma patients. Moreover, high HYAL2 expression can be used as a key indicator for glioma patient prognosis. HYAL2 knockdown reduced glioma cell viability and induced glioma cell apoptosis and cell cycle arrest. Therefore, these results support that targeting HYAL2 in glioma may provide a new approach to eliminate glioma cells and improve the prognosis of glioma patients with further research.

## Methods and materials

### Cell culture

Glioma cell lines (U251, LN229, U87, and A172) and HUVECs were obtained from the Neurosurgery Laboratory of the First Affiliated Hospital of Harbin Medical University. Glioma cell lines were cultured in Dulbecco’s modified Eagle’s medium (DMEM; D6429, Sigma, USA) with 10% fetal bovine serum (FBS; 16000-044, Gibco, USA), and Roswell Park Memorial Institute (RPMI)-1640 medium (RPMI-1640; R8758, Sigma, USA) containing 10% FBS was utilized to culture the HUVEC cell line. Finally, these cell lines were placed in a 37 °C incubator with 5% CO_2_.

### Glioma tissue collection

Glioma tissues and traumatic brain tissues were derived from surgical resection at the Department of Neurosurgery, First Affiliated Hospital of Harbin Medical University. The Ethics Committee of the First Affiliated Hospital of Harbin Medical University agreed to carry out this research.

### MTT assay

HYAL2 siRNA was used to treat U251 and LN229 glioma cell lines for 48 h. After 10 µl MTT (5 mg/ml) was added to each cell culture well, the cell culture dish was cultured in a 37 °C incubator for another 4 h. Then, 150 µl DMSO was used to replace the original medium. A BioTek ELx800 (USA) microplate reader was used to evaluate cell viability at 490 nm.

### EdU assay

The U251 and LN229 glioma cell lines were cultured with siHYAL2 for 48 h. Then, an EdU assay kit (Beyotime, China) was used to measure the effect of siHYAL2 on glioma proliferation viability according to the instructions.

### Colony formation assay

U251 glioma cells were routinely digested, and a cell suspension was prepared. Then, 1000 cells per well were seeded in a 6-well cell culture dish. Next, siHYAL2 was used to transfect glioma cells. Then, the cells were cultured for 10 days. Next, 4% paraformaldehyde was added to fix the cells for 15 min, and the cells were stained with crystal violet solution.

### Western blotting

U251 and LN229 cell lines were treated with siHYAL2 for 48 h. Then, the glioma cells were lysed in RIPA solution on ice for 30 min. Next, total protein was extracted from glioma cell lines. 12.5% SDS‒PAGE gels were used to separate protein samples, and then the protein samples were transferred to PVDF membranes. After routinely blocking the membranes and incubating them with the primary and secondary antibodies, the GeneGnome XRQ Imaging System (Syngene, UK) was used to observe the immunoreactions. The primary antibodies were as follows: anti-HYAL2 (DF13080, Affinity); anti-CCND1 (26939-1-AP, Proteintech); anti-CCNB1 (28603-1-AP, Proteintech); anti-Bcl-2 (60178-1-Ig, Proteintech); anti-BAX (50599-2-Ig, Proteintech); and anti-β-actin (TA-09, ZSGB-BIO).

### Cell transfection

Lipofectamine 2000 reagent (Cat# 11,668,019, Invitrogen, USA) and siRNA were formulated into a transfection working solution according to the proportion, and then it was used to transfect glioma cell lines according to the manufacturer’s instructions. HYAL2 siRNAs were obtained from GENERAL BIOSYSTEMS (China).

### qRT‒PCR

Total RNA was collected with TRIzol reagent (Cat# T9424, Sigma, USA) from glioma cell lines (U251 and LN229). The Roche Transcriptor cDNA Synthesis Kit (Cat# 4,897,030,001, Roche, Switzerland) was used to obtain cDNA. Next, the cDNA was mixed well with the SYBR Green PCR Master Mix Kit (Cat# 4,913,914,001, Roche, Switzerland) according to the instructions. Finally, an ABI Prism 7500 fast thermocycler (Applied Biosystems, CA, USA) was used to measure the expression levels of target genes. The primer sequences for HYAL2 and GAPDH are shown in Table 1.

**Table 1:** Sequence of siRNA, primer

siRNA sequence	
siNC	5′UUC UCC GAA CGU GUC ACG UTT3
HYAL2-siRNA1	5′CAC CUA AUG AGG GUU UUG UTT3
HYAL2-siRNA2	5′GCA CAA UAU GAG UUU GAG UTT3
HYAL2-siRNA3	5′GCU ACA AUC AUG AUU AUG UTT3
Primer sequence	
GAPDH	F-5′ CACCCACTCCTCCACCTTTGA3′, R-5′ACCACCCTGTTGCTGTAGCCA3′
HYAL2	F-5’GGCCCCACCGTTACATTGG 3′, R-5’ATTCTGGTTCACAAAACCCTCAT3′

### IHC

The collected glioma tissues were embedded in paraffin. The subsequent processes were performed as reported previously. The primary antibody used for immunohistochemical staining was anti-HYAL2 (DF13080, Affinity).

### Flow cytometry

Glioma cells were treated with siHYAL2 for 48 h. Next, the collected cells were stained with the Cell Cycle and Apoptosis Analysis Kit (Beyotime, China) or the Annexin V-FITC Apoptosis Detection Kit (Beyotime, China) according to the instructions. Finally, the observations were obtained by flow cytometry.

### Bioinformatics analysis and statistical analysis

The pancancer transcriptome dataset that included 33 kinds of cancer transcriptome data was downloaded from The Cancer Genome Atlas (TCGA) database. The glioma transcriptome and clinical data were derived from the TCGA and CGGA databases. The online database GEPIA was used to obtain the expression data of HYAL1, HYAL2, HYAL3, HYAL4, HYALP1 and SPAM1 in glioma. Prism software version 7.0 was used to analyze the differences between groups. Bioinformatics analysis was completed by R software (version-4.2.0). The R packages used for bioinformatics analysis were derived from http://bioconductor.org/. The different numbers of asterisks indicate p < 0.05, < 0.01, and < 0.001 in pictures.

## Results

### Hyaluronidase (HAase) exhibits different expression patterns in pancancer and glioma

**Fig. 1:**
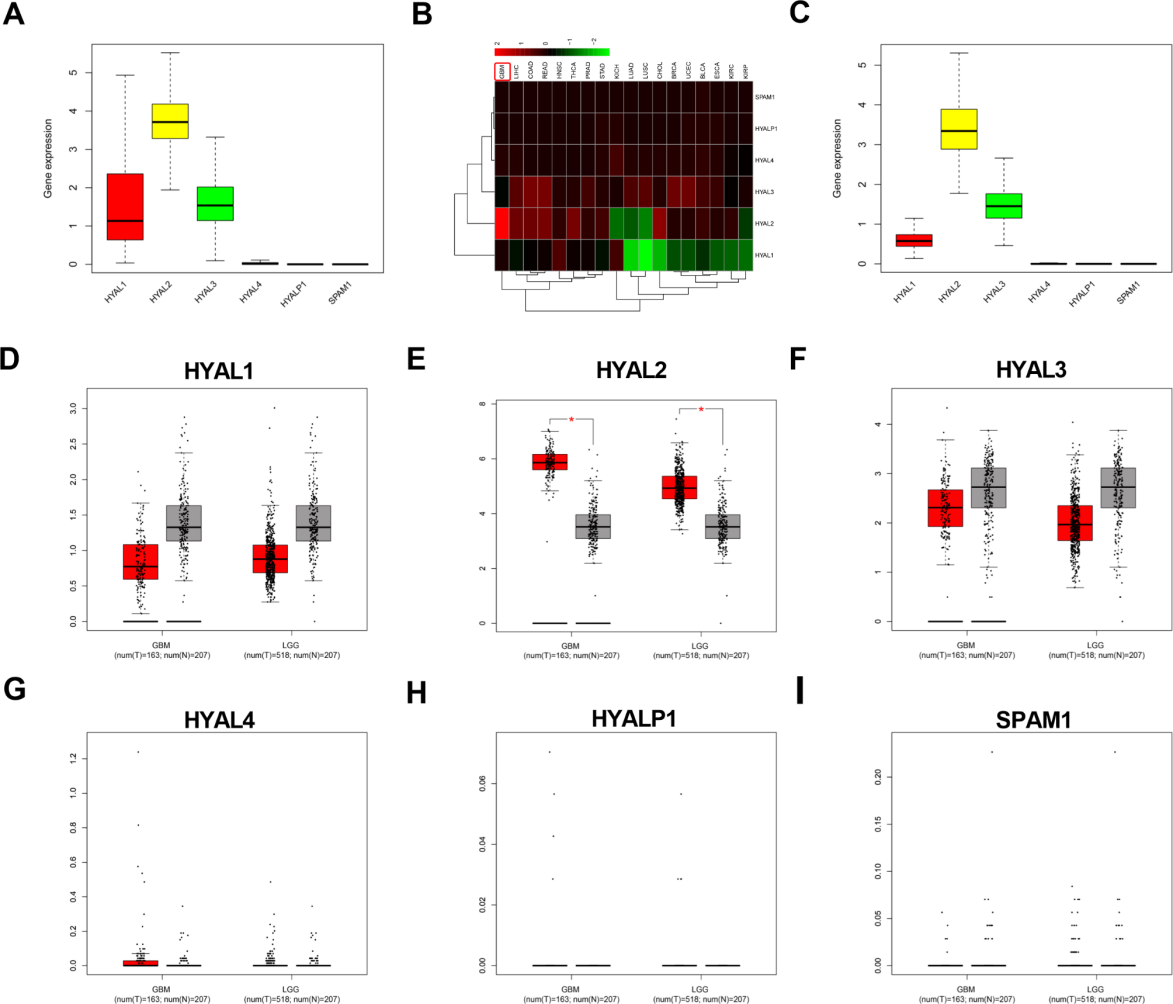
The expression pattern of hyaluronidase (HAase) in pancancer and glioma. (A) The expression levels of HYAL1, HYAL2, HYAL3, HYAL4, HYALP1 and SPAM1 in pan-cancer samples from the TCGA database. (B) The heatmap of HYAL1, HYAL2, HYAL3, HYAL4, HYALP1 and SPAM1 in pan-cancer samples from the TCGA database. (C) The expression levels of HYAL1, HYAL2, HYAL3, HYAL4, HYALP1 and SPAM1 in glioma samples from the TCGA database. (D-I) The expression levels of HYAL1, HYAL2, HYAL3, HYAL4, HYALP1 and SPAM1 in glioma samples from the GEPIA database. Bars represent the mean ± SD; *P < 0.05, **P < 0.01, and ***P < 0.001

In a previous study, we found that abnormal HA metabolism is associated with glioma malignant progression, and interfering with HA synthesis has an inhibitory effect on glioma [10]. In the present study, we aimed to explore the effect of HA degradation on glioma. In humans, the six members of the HAase family (HYAL1, HYAL2, HYAL3, HYAL4, HYALP1, SPAM1) are related to HA degradation [14]; therefore, the expression level of HAases was discussed in this research. First, the expression pattern of HAases in different tumors was explored in the The Cancer Genome Atlas (TCGA) database, HYAL2 showed the highest expression levels among six kinds of HAases, and the expression of HYAL2 was increased in GBM compared with other tumors (Fig. 1A-B). Next, to unlock the HAase expression pattern in glioma, TCGA and Gene Expression Profiling Interactive Analysis (GEPIA) databases were used to quantify HAase mRNA expression in glioma. We found that among the six HAases, only HYAL2 expression was markedly overexpressed in glioma, and the HYAL2 expression was statistically significant in glioma relative to normal brain tissue (Fig. 1C-I). These results supported that in pan-cancer and glioma, HAase has a different expression pattern and HYAL2 was overexpressed.

### HYAL2 expression is abnormally high in glioma and is negatively correlated with glioma prognosis

**Fig. 2:**
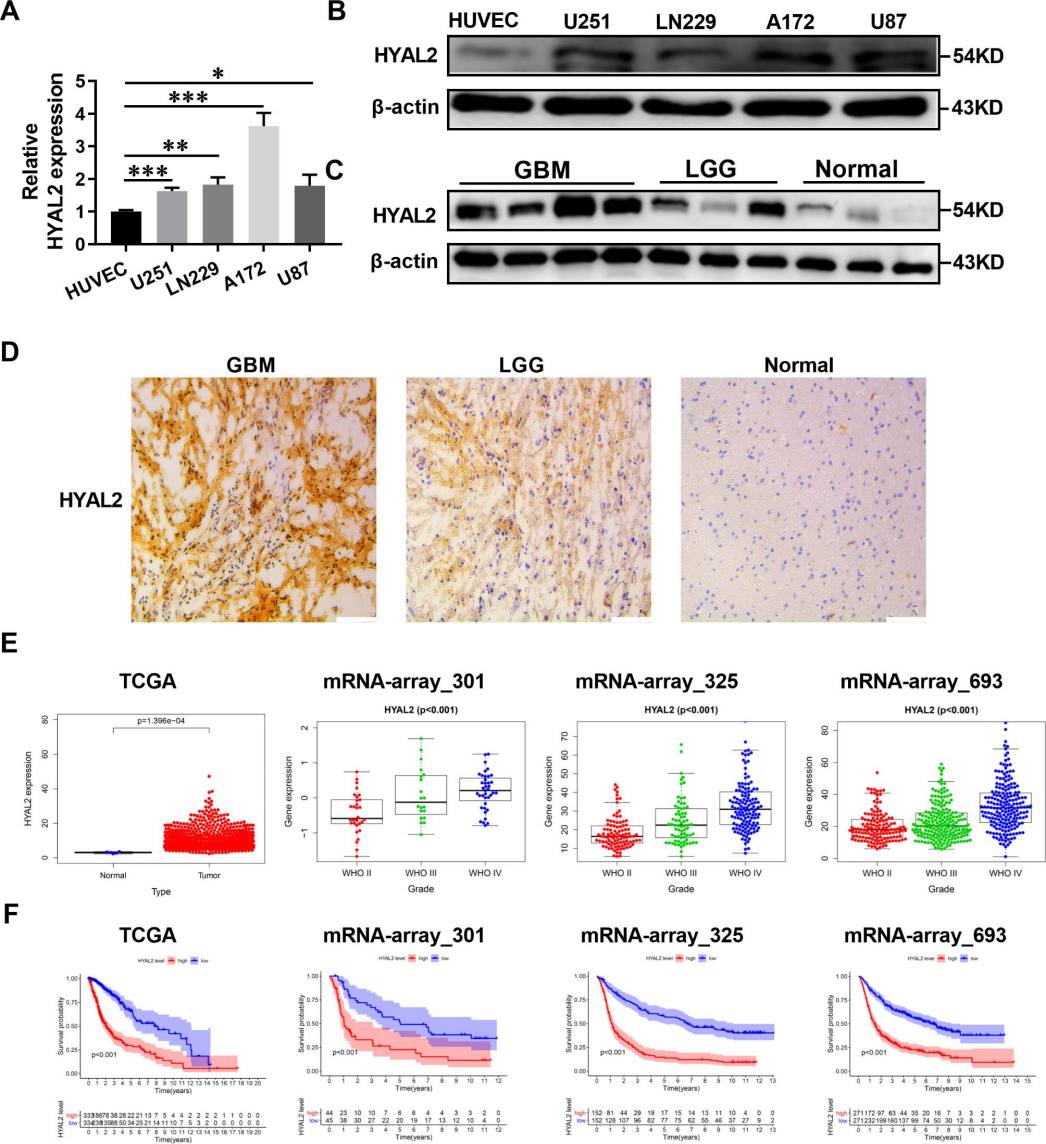
HYAL2 is overexpressed and associated with a poor prognosis in glioma. (A) The relative HYAL2 expression levels in HUVEC, U251, LN229, A172 and U87 cell lines were measured by qRT‒PCR. (B) The HYAL2 protein relative levels in HUVEC, U251, LN229, A172 and U87 cell lines were detected by Western blotting. (C) The HYAL2 protein relative levels in different grade glioma and normal brain tissues were detected by Western blotting. (D) Representative IHC staining of HYAL2 in different grades of glioma and normal brain tissues. Scale bar: 100 μm (E) The relative HYAL2 expression levels in glioma from TCGA and CGGA (mRNA-array_301, mRNAseq_325 and mRNAseq_693) databases. (F) The correlation of HYAL2 expression with glioma patient survival from TCGA and CGGA (mRNA-array_301, mRNAseq_325 and mRNAseq_693) databases. Bars represent the mean ± SD; *P < 0.05, **P < 0.01, and ***P < 0.001

Due to HYAL2 is overexpressed in multiple cancers including glioma, we aimed to further demonstrate the correlation of HYAL2 with glioma. First, the differentially expressed genes (DEGs) in glioma tissues relative to normal tissues in the TCGA database were assessed. Then, these DEGs were intersected with HAase genes (HYAL1, HYAL2, HYAL3, HYAL4, HYALP1, SPAM1), HYAL2 was the only overlapping gene (Supplementary Fig. 1A). In the Chinese Glioma Genome Atlas (CGGA) database, survival filtration analysis was performed to find the DEGs in mRNA-array_301, mRNA-seq_325 and mRNA-seq_693; next, intersection analysis was used to identify overlapping genes between DEGs screened in mRNA-array_301, mRNA-seq_325 and mRNA-seq_693 and HAase genes (HYAL1, HYAL2, HYAL3, HYAL4, HYALP1, SPAM1), and again, HYAL2 was the only overlapping gene (Supplementary Fig. 1B). These evidences supported that HYAL2 was abnormally overexpressed in glioma. Therefore, we assessed the relative mRNA expression level of HYAL2 in four glioma cell lines (U251, LN229, U87, A172) and HUVECs, which showed that the expression of HYAL2 in glioma cell lines was increased significantly compared with that in HUVECs (Fig. 2A). Western blot analysis revealed that the relative protein expression levels of HYAL2 were higher than those in HUVECs (Fig. 2B). In human specimens, the Western blotting and immunohistochemical staining results indicated that the protein expression levels of HYAL2 were increased in glioma tissues relative to normal brain tissues and it was positively correlated with glioma grade (Fig. 2C-D). To demonstrate the correlation of HYAL2 expression levels with glioma prognosis, TCGA and CGGA databases were used to analyze this relationship. Then, the relative expression of HYAL2 was quantified based on TCGA and CGGA datasets (mRNA-array_301, mRNAseq_325 and mRNAseq_693). HYAL2 expression was increased in glioma tissues compared with normal tissues in the TCGA database; moreover, in the CGGA database, the expression of HYAL2 was positively correlated with the grade of glioma (Fig. 2E). We next explored the relationship between HYAL2 expression and glioma patient survival time, and Kaplan‒Meier survival analysis demonstrated that glioma patients with high HYAL2 expression had a shorter survival time in the TCGA and CGGA datasets (mRNA-array_301, mRNAseq_325 and mRNAseq_693) (Fig. 2F). Taken together, these data supported that HYAL2 expression was abnormally increased in glioma and negatively correlated with glioma prognosis.

### High HYAL2 expression may act as an independent risk indicator in glioma patients

**Fig. 3:**
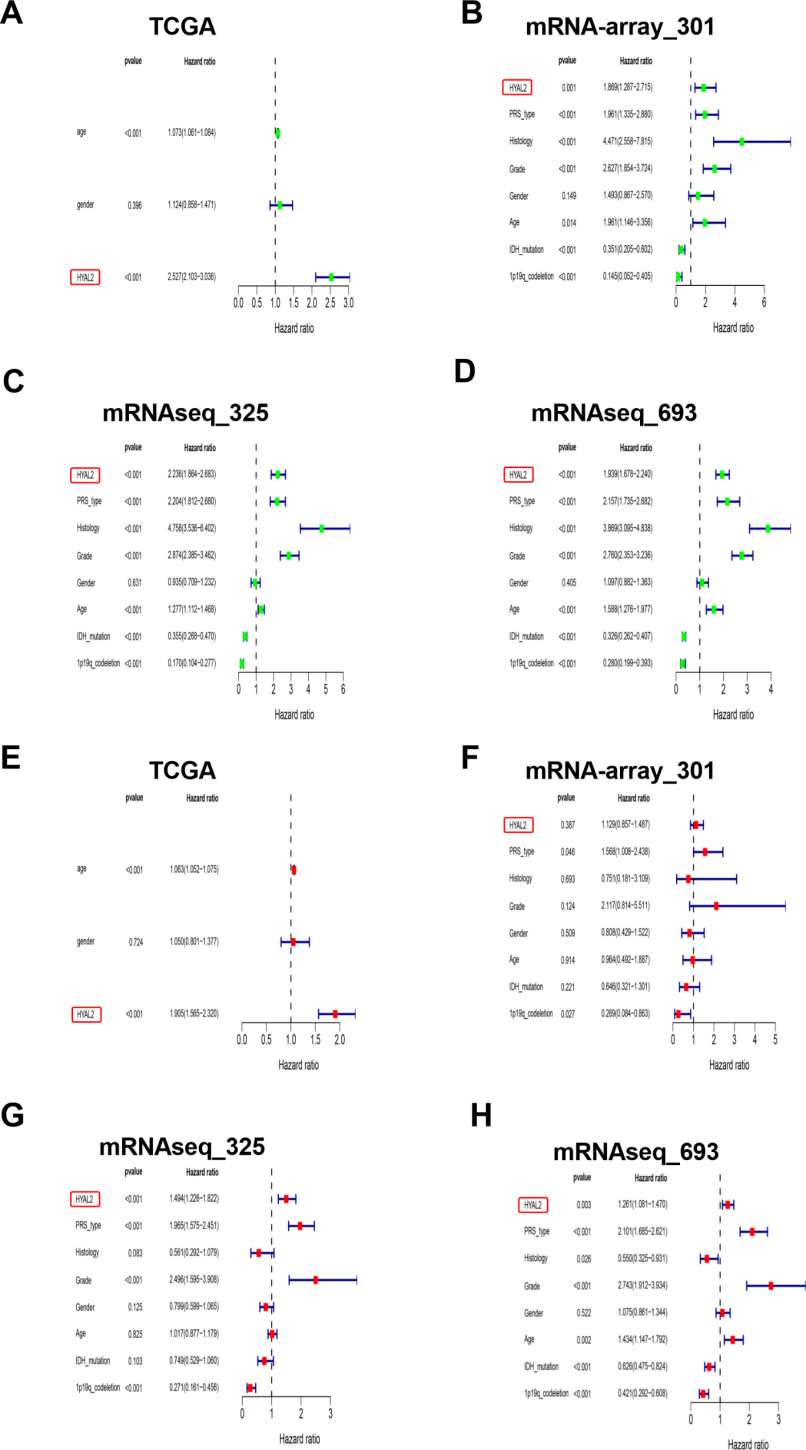
HYAL2 overexpression is a crucial high-risk indicator in glioma. (A-D) Univariate Cox analysis of HYAL2 with glioma hazard ratio (HR) in TCGA and CGGA (mRNA-array_301, mRNAseq_325 and mRNAseq_693) datasets. (E-H) Multivariate Cox analysis of HYAL2 with glioma HR in the TCGA and CGGA (mRNA-array_301, mRNAseq_325 and mRNAseq_693) databases. Bars represent the mean ± SD; *P < 0.05, **P < 0.01, and ***P < 0.001

To elucidate the feasibility of HYAL2 serving as an independent risk factor for glioma prognosis, the univariate Cox analysis demonstrated that HYAL2 expression levels were negatively associated with glioma prognosis in the TCGA database (HR = 2.527, P < 0.001) (Fig. 3A). Moreover, as shown in Fig. 3B-D, similar results were obtained in the CGGA database (mRNA-array_301: HR = 1.869, P = 0.001; mRNAseq_325: HR = 2.236, P < 0.001; mRNAseq_693: HR = 1.939, P < 0.001). These results preliminarily demonstrated that increased HYAL2 expression may be an independent risk factor in glioma patients. To further investigate the correlation of HYAL2 with glioma prognosis, multivariate Cox analysis was used to uncover this correlation. As shown in Fig. 3E-H, the high expression of HYAL2 could act a risk indicator in the TCGA (HR = 1.905, P < 0.001) and CGGA datasets (mRNA-array_301: HR = 1.129, P = 0.387; mRNAseq_325: HR = 1.494, P < 0.001; mRNAseq_693: HR = 1.261, P = 0.003); however, multivariate Cox analysis of the CGGA dataset mRNA-array_301 did not reveal strong statistical significance. A possible explanation was that multivariate Cox analysis needs to integrate multiple clinical variables for analysis; however, in the CGGA (mRNA-array_301) database, some patients were excluded from the analysis due to incomplete clinical information, and fewer patients were analyzed, ultimately leading to the occurrence of this phenomenon. Overall, these univariate and multivariate Cox analysis data suggested that increased HYAL2 expression may represent an independent risk factor for glioma patient prognosis.

### Increased HYAL2 is associated with multiple clinical traits in glioma patients

**Fig. 4:**
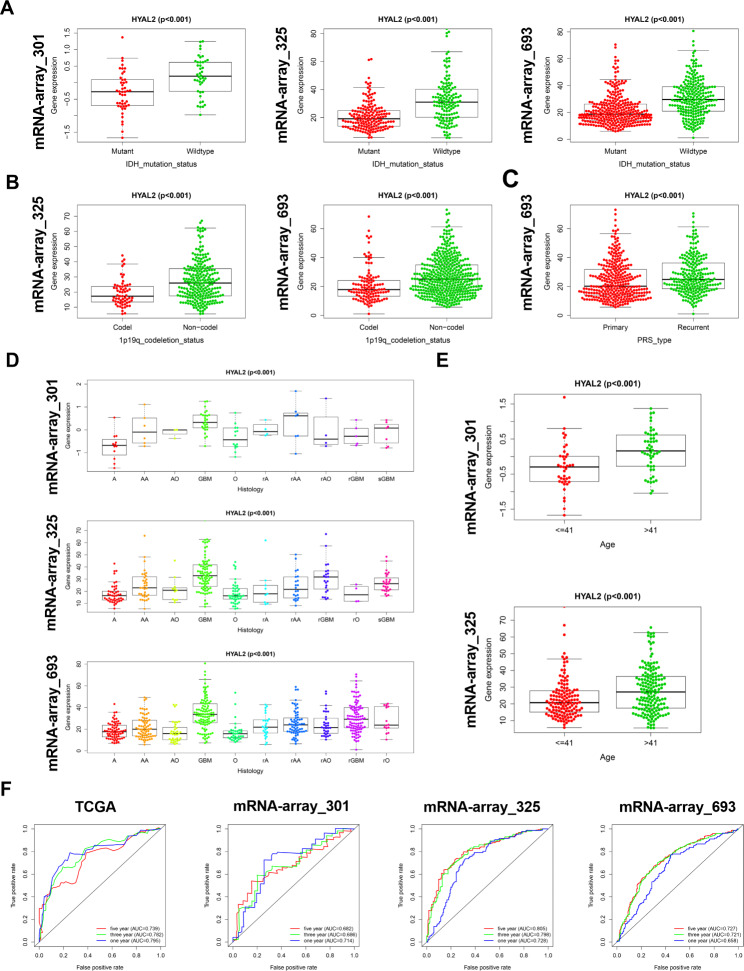
HYAL2 overexpression is related to multiple clinical traits in glioma patients. (A) The relationship between HYAL2 expression and glioma IDH mutation status in the CGGA (mRNA-array_301, mRNAseq_325 and mRNAseq_693) database. (B) The relationship between HYAL2 expression and glioma 1p19q codeletion status in the CGGA (mRNAseq_325 and mRNAseq_693) database. (C) The relationship between HYAL2 expression and glioma recurrence status in the CGGA (mRNAseq_693) database. (D) The relationship between HYAL2 expression and glioma histology in the CGGA (mRNA-array_301, mRNAseq_325 and mRNAseq_693) database. (E) The relationship between HYAL2 expression and glioma patient age in the CGGA (mRNA-array_301 and mRNAseq_325) database. (F) The ROC curve for predicting the one-year, three-year and five-year survival outcomes based on HYAL2 expression in glioma patients. Bars represent the mean ± SD; *P < 0.05, **P < 0.01, and ***P < 0.001

Because HYAL2 was overexpressed in glioma and closely related to a poor prognosis in glioma patients, we further investigated the correlation of HYAL2 expression with the clinical characteristics of glioma patients. In the CGGA (mRNA-array_301, mRNAseq_325 and mRNAseq_693) database, the data regarding HYAL2 mRNA expression and the patients’ clinical information, including age, histology and IDH mutation status and 1p19q codeletion status, were extracted. The correlation analysis demonstrated that the expression of HYAL2 was closely associated with the IDH mutation status and 1p19q codeletion status (Fig. 4A-B). As shown in Fig. 4C, HYAL2 expression was higher in recurrent glioma than in primary glioma. Moreover, in terms of glioma histology classification, HYAL2 overexpression was closely associated with the histology of glioma (Fig. 4D). In the mRNA-array_301 and mRNAseq_325 datasets, with increasing patient age, the expression level of HYAL2 also increased (Fig. 4E). In addition, to explore the clinical diagnostic value of HYAL2 for glioma, receiver operating characteristic (ROC) curves were generated to evaluate the accuracy of HYAL2 as a glioma prognostic factor in TCGA and CGGA (mRNA-array_301, mRNAseq_325 and mRNAseq_693) datasets. In the ROC curve, the AUC value corresponds to the area under the ROC curve, and an AUC greater than or equal to 0.7 indicates that a given variable can accurately predict prognosis. As shown in Fig. 4F, in the TCGA and CGGA databases, the AUC values of ROC in predicting the one-year, three-year and five-year survival outcomes of glioma patients were all greater than 0.7 or close to 0.7, suggesting that high expression of HYAL2 can act as an indicator for predicting the survival outcome of glioma patients. Briefly, all of these results revealed that high expression of HYAL2 was closely associated with multiple malignant clinical characteristics of glioma and also serves as a predictive factor for glioma prognosis.

### Targeting HYAL2 exhibits an anti-glioma effect

**Fig. 5:**
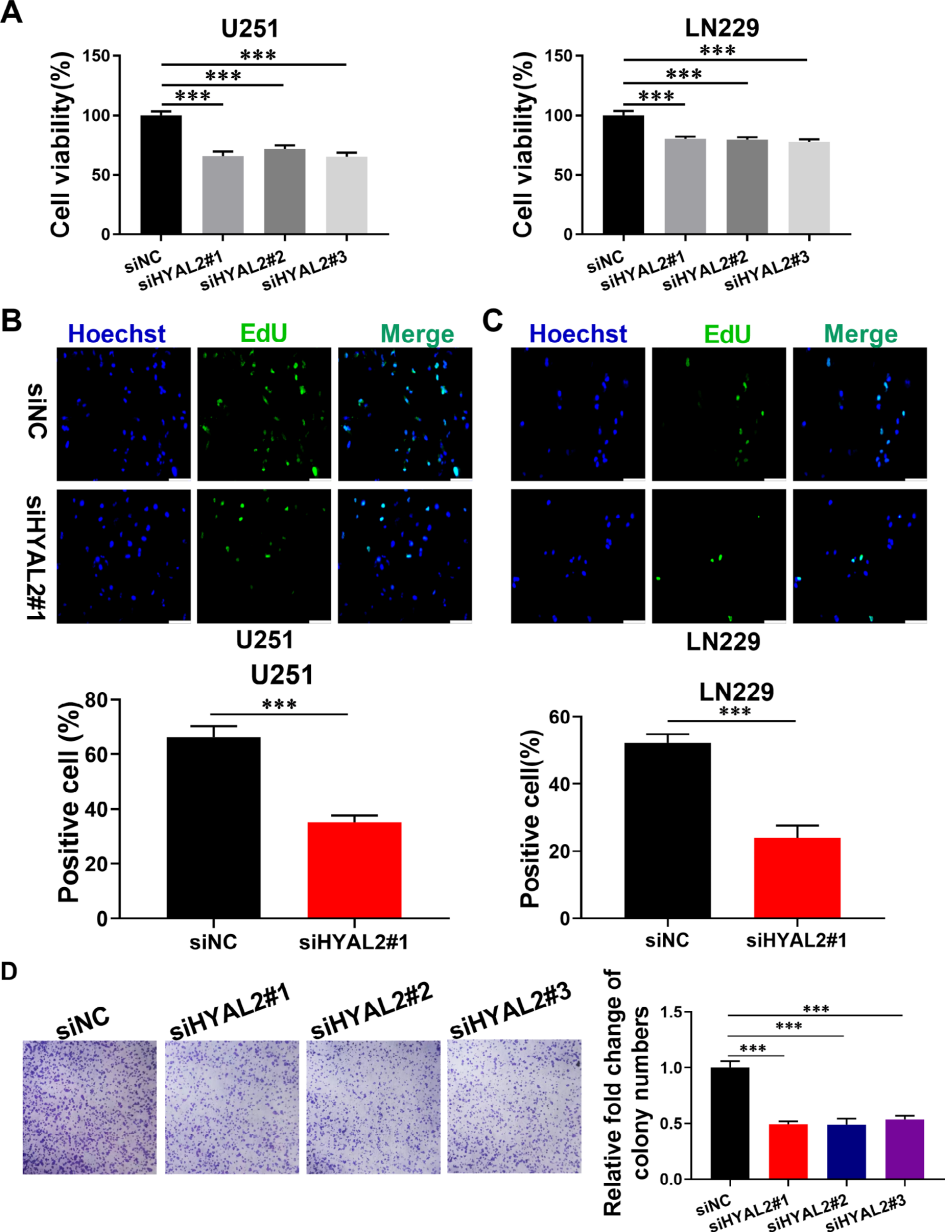
HYAL2 knockdown has a satisfactory therapeutic outcome in glioma. (A) The viability of U251 and LN229 cell lines treated with siHYAL2 for 48 h was detected by MTT assay. (B-C) The proliferation viability of U251 and LN229 cell lines treated with siHYAL2 for 48 h detected by EdU assay. Scale bar: 100 μm (D) The proliferation viability of U251 treated with siHYAL2 detected by colony formation assay. Bars represent the mean ± SD; *P < 0.05, **P < 0.01, and ***P < 0.001

Given that HYAL2 might act as a risk factor for glioma prognosis, we wanted to determine whether disturbing HYAL2 expression could exhibit a therapeutic outcome in glioma. Subsequently, siRNA was used to knockdown HYAL2 in U251 and LN229 glioma cell lines (Supplementary Fig. 1C). The MTT assay results showed that the viability of U251 and LN229 glioma cells was decreased significantly by siHYAL2 (Fig. 5A). After that, an EdU assay was performed to assess the glioma proliferation viability treated with siHYAL2. We found that the green fluorescence intensity could be strongly decreased by siHYAL2 in glioma, suggesting that HYAL2 knockdown could decrease glioma proliferation (Fig. 5B-C). To further determine the inhibitory effect of siHYAL2 on glioma, the results of the colony formation assay demonstrated that the inhibition of HYAL2 expression in glioma induced a reduction in the size and number of cell colonies relative to controls (Fig. 5D). These observations indicated that targeting HYAL2 could unlock a therapeutic effect for glioma.

### Interfering with HYAL2 induces cell cycle arrest and apoptosis in glioma

**Fig. 6:**
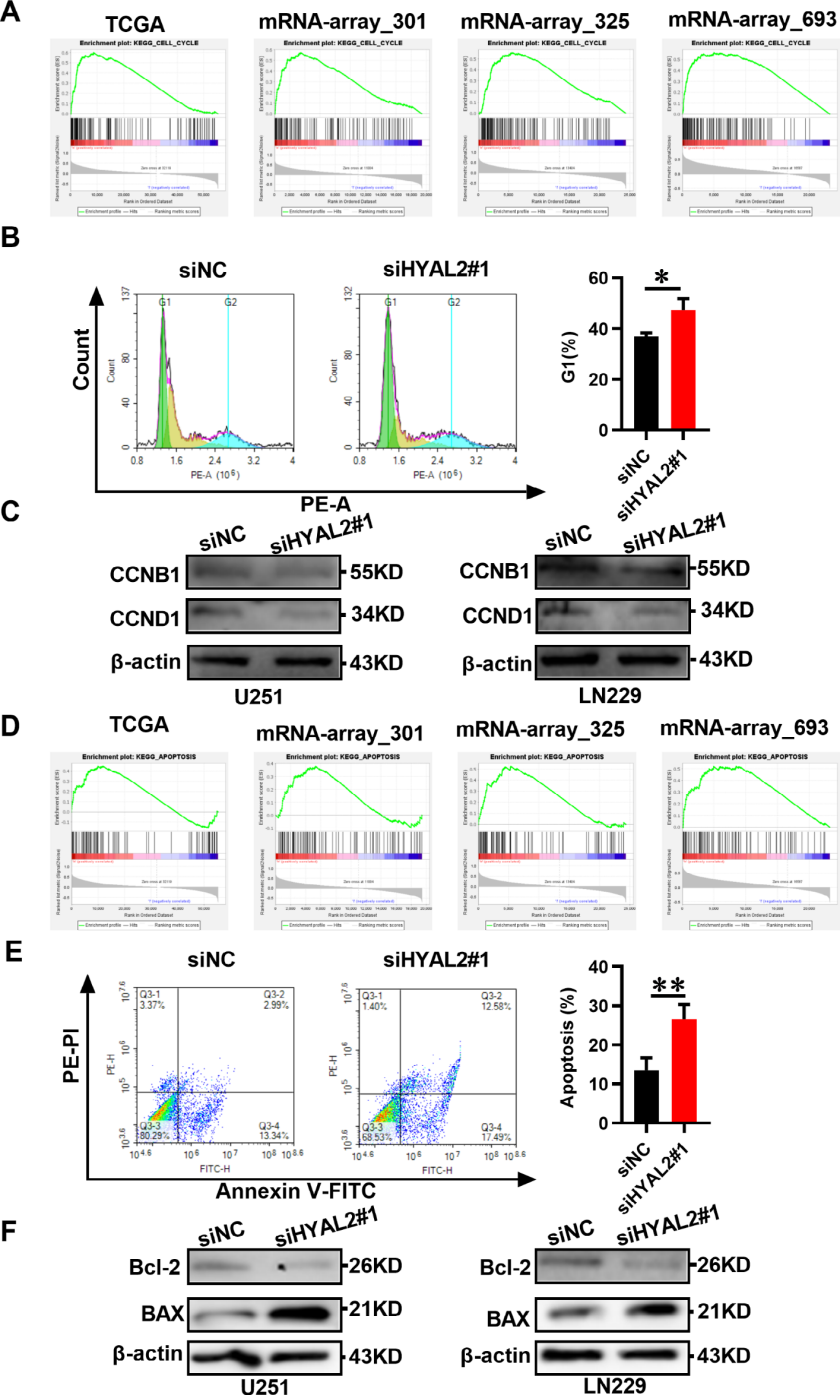
HYAL2 knockdown induces glioma cell cycle arrest and apoptosis. (A) GSEA indicated that HYAL2 expression was related to cell cycle regulation in glioma. (B) The cell cycle of U251 cells was detected by flow cytometry after treatment with HYAL2 siRNA for 48 h. (C) The relative expression of CCNB1 and CCND1 proteins in U251 and LN229 cell lines was detected by Western blotting after treatment with HYAL2 siRNA for 48 h. (D) GSEA indicated that HYAL2 expression was related to apoptosis regulation in glioma. (E) Apoptosis in U251 cells was detected by flow cytometry after treatment with HYAL2 siRNA for 48 h. (F) The relative expression of Bcl-2 and BAX proteins in U251 and LN229 cell lines was detected by Western blotting after treatment with HYAL2 siRNA for 48 h. Bars represent the mean ± SD; *P < 0.05, **P < 0.01, and ***P < 0.001

To explore the underlying mechanism by which targeting HYAL2 inhibits glioma progression, GSEA was used to identify the possible pathway affected by HYAL2. In the TCGA and CGGA databases (mRNA-array_301, mRNAseq_325 and mRNAseq_693), the GSEA results showed that high HYAL2 expression resulted in significant enrichment of the cell cycle and apoptosis pathways in glioma (Fig. 6A, D). Then, we demonstrated that HYAL2 knockdown could affect the cell cycle and apoptosis in glioma. SiHYAL2 was utilized to treat the U251 glioma cell line, and flow cytometry was performed to detect the effect of HYAL2 knockdown on the glioma cell cycle. We found that siHYAL2 treatment could lead to glioma cell cycle arrest in the G1 phase (Fig. 6B). Moreover, the Western blotting results showed that HYAL2 knockdown decreased the relative levels of the cell cycle-associated proteins CCNB1 and CCND1 in glioma cells, further suggesting that silencing HYAL2 could induce cell cycle arrest in glioma (Fig. 6C). After that, the apoptosis levels of glioma transfected with siHYAL2 were examined via flow cytometry, and it was found that siHYAL2 treatment could lead to an increase in the apoptosis rate of U251 glioma cells compared with that in controls (Fig. 6E). Western blotting results indicated that HYAL2 knockdown induced Bcl-2 downregulation, an anti-apoptotic protein, in glioma cells; more importantly, HYAL2 silencing increased the level of BAX, a pro-apoptotic protein, in glioma cells (Fig. 6F). These results revealed that inhibition of HYAL2 induced glioma cell apoptosis. In summary, the above results indicated that HYAL2 knockdown could exhibit an inhibitory effect on glioma progression by inducing cell cycle arrest and apoptosis.

### Identification of small molecular drugs targeting HYAL2 for glioma treatment by CMap analysis

**Fig. 7:**
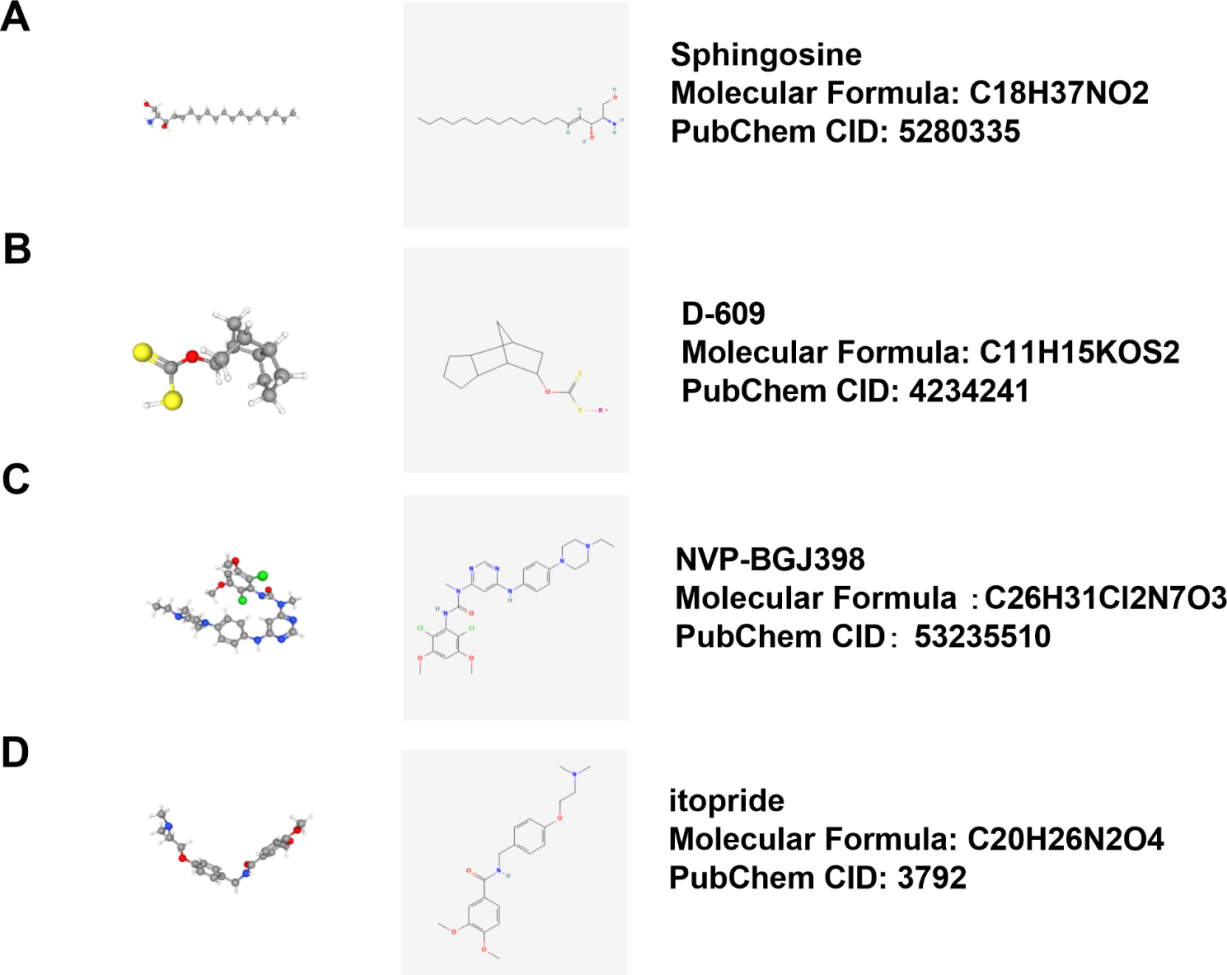
CMap analysis. (A-D) The 3D structure, 2D structure, molecular formula and PubChem CID of the four drugs predicted by CMap analysis

To identify small molecular drugs targeting HYAL2, Pearson correlation analysis was carried out to analyze the co-expressed genes with HYAL2 in the TCGA database. Finally, 20 genes positively or negatively correlated with HYAL2 expression were found (Supplementary Fig. 1D). Then, these 20 genes were uploaded to CMap (https://clue.io/connectopedia/), and ultimately, four small molecule compounds that may target HYAL2 in gliomas were discovered: sphingosine, D-609, NVP-BGJ398 and itopride. The 2D and 3D structures of four small molecule compounds were obtained from PubChem (Fig. 7A-D).

## Discussion

HA is the major macropolysaccharide in the ECM, and it is closely related to the malignant biological function of tumors. HA metabolism is abnormal in a variety of tumors and can modulate cell proliferation, motility and malignant invasive properties [15]. HA metabolism is dependent on the regulation of hyaluronic acid synthase, hyaluronic acid receptors and hyaluronidase [16]. Our previous study confirmed that the expression of hyaluronan synthase 3 (HAS3) and CD44 is elevated in gliomas; on the one hand, increased HAS3 expression can promote HA biosynthesis, and on the other hand, overexpressed CD44 can combine with HA to accelerate glioma progression [10]. Moreover, inhibition of the HA pathway can induce M1-like macrophage polarization in GBM [11]. Given that our previous study mainly focused on the effect of HA synthesis in gliomas, in this study, we preliminarily explored the role of hyaluronidases in glioma progression. We found that HYAL2 expression in gliomas was abnormally high and closely related to the prognosis of glioma patients.

HA catabolism is mediated by the hyaluronidase family, and six hyaluronidase genes (HYAL1, HYAL2, HYAL3, HYAL4, HYALP1, and SPAM1) have been identified in humans [14]. Increasing evidence has confirmed that HAase is a crucial determinant of malignant tumor characteristics, including growth, metastasis, and angiogenesis, and exhibits different expression patterns in different tumors [17]. HYAL1 is observably overexpressed and can also be utilized as a predictor of recurrence in prostate cancer [18]. The activity of multiple hyaluronidase isoforms (HYAL1, 2, 3, and SPAM1) is increased in colon cancer patients, and the increased HYAL1 and HYAL2 are particularly closely related to the aggressiveness of cancer [19]. Compared with that in healthy tissue, the expression of HYAL2 was significantly decreased in endometrial cancer; in contrast, the expression in breast cancer was markedly increased, especially at the margins of invasive breast cancer [19–21]. In this research, we clearly confirmed that the expression of HYAL2 is abnormally increased in glioma samples in the TCGA, CGGA databases and glioma specimens, and that the HYAL2 expression level is significantly negatively correlated with the glioma patient’s survival time. Subsequent univariate, multivariate Cox and ROC analysis results showed that the expression level of HYAL2 could act as a prognostic factor for glioma, and it was also correlated with a variety of clinical traits in glioma patients, including histology, IDH status, and 1p19q codeletion status. These results all suggested that increased expression of HYAL2 in gliomas is significantly correlated with a poor prognosis in glioma patients, which preliminarily supports that HYAL2 may be a therapeutic target for gliomas.

To assess the potential therapeutic value of targeting HYAL2, HYAL2 siRNA was used to treat glioma, and it was found that HYAL2 knockdown reduced glioma cell viability and proliferation. We further explored the possible signaling pathway by which HYAL2 affects glioma by GSEA, meanwhile, found that HYAL2 could significantly affect apoptosis and the cell cycle in glioma. Subsequent studies demonstrated that targeting HYAL2 can induce glioma cell apoptosis and arrest the cell cycle in the G1 phase. In addition, it has been reported that cell cycle signals can modulate cell proliferation [22]. Considering these findings, we hypothesized that targeting HYAL2 could induce apoptosis and inhibit glioma cell proliferation by arresting the cell cycle in G1 phase, ultimately restraining glioma growth.

HA is a relatively simple polysaccharide, and its family mainly includes high molecular weight HA (HMW-HA), low molecular weight HA (LMW-HA), and oligomeric HA (o-HA), which are mainly achieved by the tight coordination of HA biosynthesis and degradation processes [16]. Moreover, HA has different molecular functions according to its molecular weight. For instance, HMW-HA is able to inhibit angiogenesis and is considered to inhibit tumor growth, while LMW-HA can induce inflammation and promote angiogenesis, and its abnormal accumulation is involved in malignant tumor behaviors. o-HA can inhibit glioma growth by inducing glioma apoptosis and downregulating critical survival genes [14, 23, 24]. Thus, LMW-HA is essential for tumor cell survival. Consistent with LMW-HA accumulation in cancer, hyaluronidase expression is abnormally increased in many tumors, including colon, bladder, prostate, brain, and breast tumors [14]. Our previous results confirmed that HAS3 overexpression could accelerate HA synthesis in glioma and thus promote glioma malignant progression. In addition, in this study, we demonstrated that HYAL2 was unusually highly expressed in glioma. HA synthases (HASs) mainly catalyze the synthesis of HMW-HA [23]. Mechanistically, HMW-HA can be anchored on the cell surface by CD44 and is hydrolyzed by HYAL2 into HA small molecules [25]. Therefore, we speculated that it is highly likely that the overexpression of HAS3 promotes the abnormal biosynthesis of HA, and then excess accumulated HMW-HA can be further degraded into LMW-HA by HYAL2, which ultimately accelerates the malignant progression of glioma. In view of this conclusion, in our next study, we will further investigate the mechanism by which HYAL2-mediated HA degradation promotes glioma progression.

Tumor-derived HAase may be an attractive therapeutic target for tumors. Many synthetic or natural products, including high molecular mass poly(styrene-4-sulfonate), fenoprofen, glycerrhizic acid, and plant-derived compounds, have been used as HAase inhibitors [17]. In the present study, Cmap, an online database, was utilized to predict possible small molecule compounds targeting HYAL2. Finally, four small molecule compounds were discovered: sphingosine, D-609, NVP-BGJ398 and itopride. Although the roles of these four small molecule compounds in glioma have not yet been reported, sphingosine 1-phosphate (S1P) has been highlighted as a key regulator of tumor progression, and ceramidase inhibitors are able to limit ceramide conversion into sphingosine and fatty acids, ultimately inhibiting sphingosine 1-phosphate (S1P) biosynthesis [26, 27]. This evidence highlights that sphingosine, as a ceramidase inhibitor, has the potential to inhibit tumor progression by inhibiting ceramide conversion. D609 exerted a therapeutic effect in a non-small cell human lung carcinoma (NSCLC) xenograft model [28]. Moreover, D609 could cause human tumor cell death; in contrast, an effect on normal cells was not discovered [29]. The FDA approved infigratinib (NVP-BGJ398), an FGFR2 inhibitor, for the treatment of patients with advanced and metastatic cholangiocarcinoma [30]. In addition, dopamine receptors (DRs) have been found in many tumors; recently, some studies reported that DR antagonists have the ability to enhance the sensitivity of cancer stem cells to chemotherapy, and in mouse models, the dopamine receptor antagonist trifluoperazine also exhibits an inhibitory effect on GBM [31, 32]; therefore, these reports implied that itopride, as a dopamine receptor antagonist, has the ability to restrain tumor growth. Additionally, the reliability of CMap as a potential drug prediction tool for the disease has been confirmed, and in the next study, we will focus on the effects of these small molecule drugs as HYAL2 inhibitors in glioma. Overall, we think that these four compounds may become potential drugs for adjuvant chemotherapy in glioma with in-depth research in the future.

In conclusion, our observations revealed that HYAL2 overexpression in gliomas is associated with prognosis. Together with our previous studies, all of these results suggest that there are metabolic abnormalities related to HA in gliomas, including abnormalities in HA biosynthesis and catabolism. Therefore, the results of this study further enhance the understanding of the role of HA in glioma pathogenesis and provide a novel target for glioma treatment.

### Electronic supplementary material

Below is the link to the electronic supplementary material.

Supplementary Material 1

Supplementary Material 2

## Data Availability

The publicly available datasets included in this research could be found in Material and Methods.
