# Supplementary material for: Inhibition of hyaluronic acid degradation pathway suppresses glioma progression by inducing apoptosis and cell cycle arrest
Source: Cancer Cell Int. 2023 Aug 11;23:163. doi: 10.1186/s12935-023-02998-4 (PMC10422813; doi:10.1186/s12935-023-02998-4)
Supplement: Supplementary file 1 — Supplementary Material 1 [file 12935_2023_2998_MOESM1_ESM.docx]

**Supplementary Figure 1**

1. The intersection analysis of differentially expressed genes in glioma from the TCGA database and HAase-related genes. (B) The intersection analysis of survival-related genes in the CGGA database (mRNA-array_301, mRNAseq_325 and mRNAseq_693) and HAase-related genes (list 1: mRNAseq_693; list 2: mRNAseq_325; list 3: mRNA-array_301; list 4: HAase-related genes). (C) Gene knockout efficiency of the HYAL2 siRNA were measured by qRT‒PCR.. (D) List of the top 20 genes with positive and negative co-expression relationships with HYAL2 in the TCGA database.
