# Supplementary figures and images for: Inhibition of hyaluronic acid degradation pathway suppresses glioma progression by inducing apoptosis and cell cycle arrest
Source: Cancer Cell Int. 2023 Aug 11;23:163. doi: 10.1186/s12935-023-02998-4 (PMC10422813; doi:10.1186/s12935-023-02998-4)

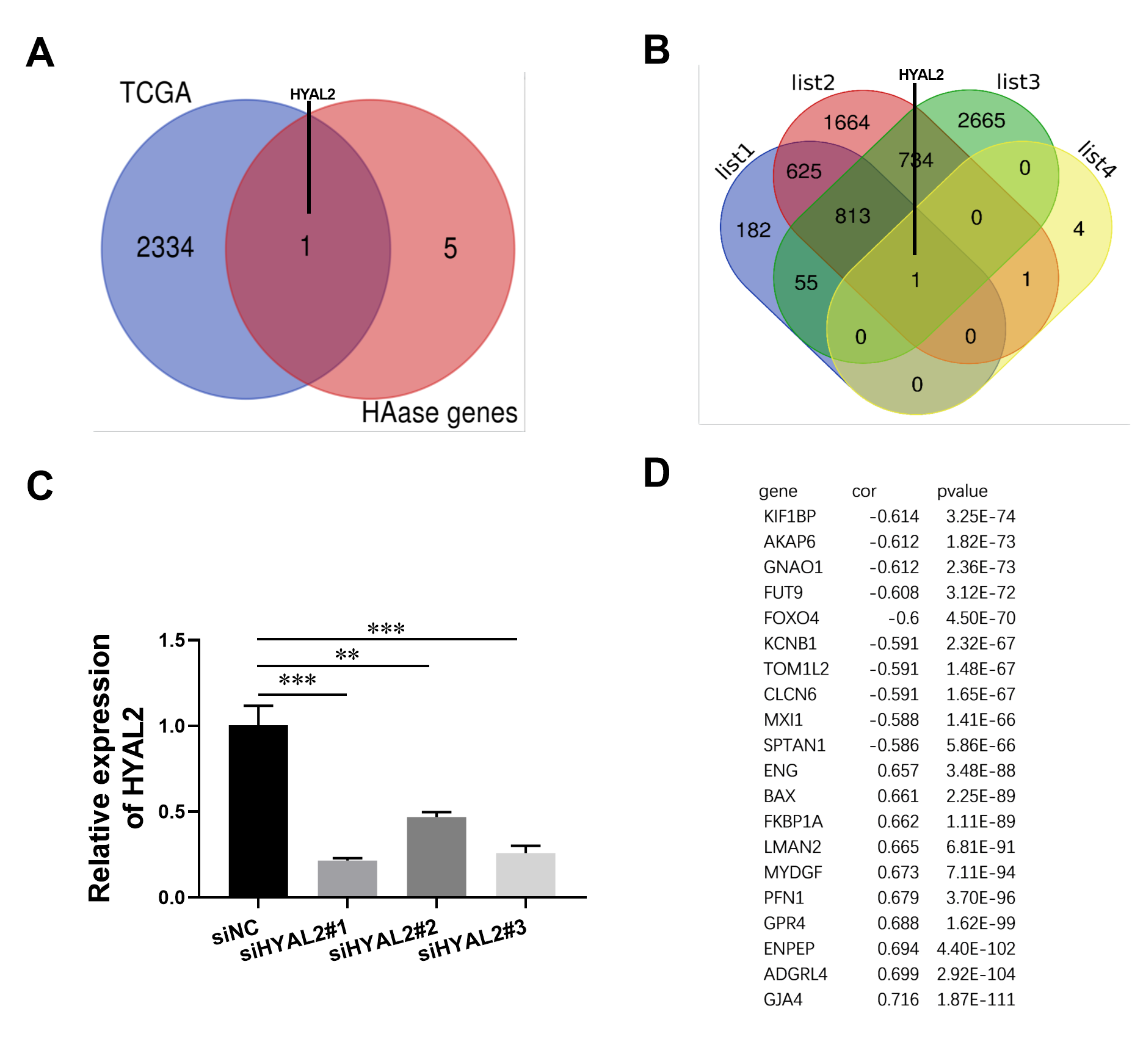

Supplement: Supplementary file 2 — Supplementary Material 2 [file 12935_2023_2998_MOESM2_ESM.png]
